# Personalized Treatment in Ovarian Cancer: A Review of Disease Monitoring, Biomarker Expression, and Targeted Treatments for Advanced, Recurrent Ovarian Cancers

**DOI:** 10.3390/cancers17111822

**Published:** 2025-05-30

**Authors:** Victoria M. Ettorre, Abdelrahman AlAshqar, Namrata Sethi, Alessandro D. Santin

**Affiliations:** Department of Obstetrics, Gynecology, and Reproductive Sciences, Yale University School of Medicine, New Haven, CT 06520, USA; victoria.ettorre@yale.edu (V.M.E.); abdelrahman.alashqar@yale.edu (A.A.); namrata.sethi@yale.edu (N.S.)

**Keywords:** ovarian cancer, biomarkers, ca125, circulating tumor DNA, BRCA, HER2, TROP2, FOLR1

## Abstract

**Background/Objectives**: Ovarian cancer is the most lethal gynecologic malignancy due to its late diagnosis, aggressive disease course, and high likelihood of recurrence. In the last few years, with the advent of high-throughput genomic methodologies, our understanding of ovarian cancer genetics and biology has grown. In this review, we discuss current monitoring techniques, as well as biomarker-directed therapies, recently developed for ovarian cancer treatment. **Methods**: The primary literature and review articles were obtained through PUBMED searches of “ovarian cancer”, “biomarkers”, “CA125”, “circulating tumor DNA”, “BRCA”, “HER2”, “TROP2”, and “FOLR1.” **Results and Conclusions**: The detection and quantification of CA125, a protein biomarker, remains the primary test used in the clinic for ovarian cancer diagnosis and monitoring. However, liquid biopsy techniques involving circulating tumor DNA, used alone or in combination with CA125, are increasingly used to enhance diagnostic accuracy and provide a more comprehensive picture of tumor genomic changes, including single-nucleotide variants, copy number variations, and epigenetic alterations. In the last few years, the use of BRCA, HER2, TROP2, and FOLR1 as biomarkers for targeted treatment has demonstrated promising results, both preclinically and clinically. The detection of BRCA1/2 mutations is routinely used as a strong predictor of response to PARP inhibitors, while HER2, TROP2, and FOLR1 expressions have emerged as primary targets for the treatment of recurrent ovarian cancer patients using novel antibody–drug conjugates (ADCs).

## Simple Summary

Ovarian cancer represents one of the deadliest forms of cancer affecting women. The development of improved treatment modalities, beyond surgery and platinum-based chemotherapy, remains an unmet medical need. In this review, we discuss some of the monitoring tools available to diagnose and manage ovarian cancer patients, as well as novel biomarkers, either in use or in an advanced stage of development, as targets for personalized ovarian cancer treatment.

## 1. Introduction

Ovarian cancer is the third most common gynecologic malignancy globally, and the second most common in developed countries [1,2]. Over 90% of ovarian malignancies arise from the epithelium, with high-grade serous ovarian carcinoma (HGSOC) being the predominant subtype [3]. Despite advances in our understanding of its pathogenesis, ovarian cancer remains the leading cause of gynecologic cancer-related mortality, primarily due to its frequent late-stage diagnosis and high likelihood of recurrence [4,5]. Risk factors contributing to the development of ovarian cancer include a higher number of lifetime ovulatory cycles and genetic mutations such as BRCA1/2 [6]. The current standard of care involves cytoreductive surgery, followed by platinum- and taxane-based chemotherapy. While this approach has improved survival outcomes, the majority of patients with advanced disease ultimately recur, highlighting the need for effective and durable treatment strategies after the development of platinum/paclitaxel resistance.

Recent advances in molecular profiling and biomarker-driven strategies have helped revolutionize ovarian cancer management. Liquid biopsy technologies have further improved early detection and risk stratification [7]. Traditional biomarkers, such as CA125 and HE4, remain integral to diagnosis and monitoring, but emerging biomarkers, including homologous recombination deficiency (HRD), PD-L1 expression, and tumor mutation burden (TMB), are reshaping therapeutic approaches [8,9,10]. Additionally, targets such as folic acid receptor alpha (FOLR1), epidermal growth factor receptor 2 (HER2), and trophoblast cell surface antigen 2 (TROP2) have demonstrated great potential as targets of antibody–drug conjugates, expanding therapeutic options beyond conventional chemotherapy [11]. This review explores the evolving role of biomarkers in shaping personalized treatment for ovarian cancer patients, with the goal of improving outcomes and advancing precision oncology.

## 2. Materials and Methods

The primary literature and review articles were obtained through PUBMED searches of “ovarian cancer”, “biomarkers”, “CA125”, “circulating tumor DNA”, “BRCA”, “HER2”, “TROP2”, and “FOLR1”. The papers evaluated were limited to those written in the English language, with a year cutoff of 2000 to the present.

## 3. Disease Monitoring

### 3.1. CA125

Cancer antigen 125 (CA125) has long been established as a cornerstone biomarker in epithelial ovarian cancer, with extensive utility in both disease monitoring and prognostication [12]. Clinically, CA125 serves as an early indicator of treatment failure during first-line therapy, facilitates the detection of recurrence, and guides therapeutic decisions during relapse, particularly in determining whether to continue or modify ongoing treatment regimens [13].

To standardize the clinical interpretation of CA125, the Gynecologic Cancer InterGroup (GCIG) defined the criteria for recurrence as either a confirmed doubling above the upper limit of normal (typically > 35 U/mL), or a doubling above the patient’s nadir when below the normal range, with confirmation via a second elevated measurement at least one week later [14]. Numerous studies have demonstrated that CA125 trends strongly correlate with disease recurrence, predicting progression in approximately 90% of cases. Notably, an elevation to more than twice the upper limit of normal following initial treatment is a reliable predictor of relapse [15,16]. Even upward trends within the normal range can portend recurrence in patients otherwise in clinical remission [17].

Beyond its role in surveillance, CA125 also carries prognostic significance. In advanced-stage disease, the normalization of CA125 levels after first-line chemotherapy has been associated with improved overall and progression-free survival (PFS) [16]. A reduction to less than 50% of baseline levels after two cycles of platinum-based therapy is similarly correlated with favorable outcomes [18]. Additionally, decreasing CA125 levels following neoadjuvant chemotherapy have been shown to predict improved clinical response [19]. Several other CA125 metrics offer further prognostic insight. Time to nadir and pre-treatment levels have been independently associated with survival outcomes. For instance, the early decline of CA125 within 72 h of initiating treatment and baseline levels below 230 U/mL have each been linked to superior PFS and overall survival (OS) [20]. Preoperative CA125 levels also provide predictive value for surgical outcomes. In patients with stage III–IV disease, levels ≤ 1000 U/mL were associated with a greater likelihood of achieving optimal cytoreduction, while normal levels predicted successful interval debulking [21]. Conversely, markedly elevated levels (>535 U/mL) have been associated with an increased likelihood of lymph node metastasis, which, in turn, correlates with poorer survival [22].

A key advantage of CA125 monitoring is its ability to detect recurrence well before clinical symptoms manifest. Some studies report a median lead time of 63 days between CA125 elevation and clinical recurrence, while others suggest a window ranging from 2 to 5 months [15,17]. While this preclinical rise theoretically offers a critical opportunity for timely intervention and tailored treatment planning, whether CA125 elevation alone should trigger the initiation of second-line therapy remains controversial and should, rather, be individualized. The landmark OVO5/EORTC 55955 randomized controlled trial addressed this question by evaluating the timing of second-line chemotherapy initiation in women with epithelial ovarian cancer who had achieved a complete response following first-line platinum-based chemotherapy [23]. Participants were randomized to receive either early treatment based solely on rising CA125 levels or delayed treatment initiated upon clinical or symptomatic relapse. Although the early treatment group began chemotherapy a median of 4.8 months earlier than the delayed group, no OS benefit was observed at 2 years’ follow up. Moreover, patients in the early intervention arm reported worse quality of life, likely attributable to earlier exposure to chemotherapy and its associated toxicities [23].

In response to these findings, Krell et al. conducted a follow-up study to explore the role of shared decision making in CA125 monitoring [14]. Patients were given the option to (1) opt out of routine CA125 monitoring in the absence of clinical relapse; (2) continue monitoring, but remain blinded to the results unless requested; or (3) continue routine monitoring and receive regular updates. Of the 69 women analyzed, 80% chose to opt out of routine monitoring, while the remaining 20% continued surveillance—with 79% of that subgroup preferring to be informed of the results and 21% declining regular updates [14]. The findings of the original trial should be interpreted with caution and within the context of the critiques it has received. One key concern is that women randomized to the early treatment arm may have had undetected residual disease at baseline, as indicated by their elevated CA125 levels, which could have contributed to the lack of observed survival benefit compared to the delayed treatment group [24]. This potential confounding factor might have been mitigated by performing a surveillance computed tomography (CT) scan at study entry to rule out subclinical disease. Another critique of the trial lies in the lack of standardization in the chemotherapy regimens administered upon recurrence [24]. Only one-third of participants received both carboplatin and paclitaxel, a combination known to confer survival benefits in recurrent ovarian cancer. Additionally, a second cytoreductive surgery was not offered to patients, which, while unlikely to have influenced short-term outcomes within the study’s timeframe, may have had an impact on OS if incorporated into the treatment strategy [24]. Lastly, although the study employed the GCIG criteria for defining recurrence, it is important to note that recurrent disease can still occur in the presence of CA125 values below the established threshold, potentially limiting the sensitivity of this biomarker in certain clinical scenarios [14]. Nonetheless, these findings highlight the importance of personalized decision making in CA125 surveillance and emphasize the need to balance clinical utility with patient values and quality of life.

### 3.2. Circulating Tumor DNA

To address the limitations of CA125 surveillance—particularly its suboptimal sensitivity and specificity—circulating tumor DNA (ctDNA) has recently emerged as a promising biomarker. Since 2012, ctDNA has been investigated for its prognostic relevance in ovarian cancer and its potential utility in disease monitoring [25]. ctDNA is released into the bloodstream through tumor cell apoptosis or active secretion, and its extraction offers a real-time snapshot of the tumor’s genomic landscape, including any involved mutations, copy number variants, and epigenetic phenomena such as methylation, enabling the assessment of treatment efficacy and the development of more personalized and effective therapeutic strategies [25]. These advantages are largely attributable to ctDNA’s wider dynamic range and shorter half-life, which allow for more rapid and sensitive detection of tumor burden compared to traditional biomarkers, such as CA125 or imaging [26].

In a study by Kim et al., TP53-mut ctDNA was examined as a potential biomarker for monitoring response to treatment in high-grade serous ovarian cancer [27]. In another study by Noguchi et al., the variant allele frequency (VAF) of mutations detected in ctDNA was analyzed in 10 patients undergoing neoadjuvant chemotherapy for ovarian cancer [28]. The authors observed that the VAFs of TP53, KCAN5, and GJA8 mutations decreased following treatment in chemotherapy-sensitive cases. In contrast, increases in the VAFs of KRAS, TRPS1, and TP53 mutations were noted in resistant cases, with a significantly higher overall burden of TP53 mutations observed in the resistant cohort. These findings suggest that specific mutations, as identified through ctDNA profiling, may serve as predictive and dynamic biomarkers for treatment response in ovarian cancer. Another rare but tumor-specific ctDNA variant, the fibroblast growth factor receptor 2 (FGFR2) fusion gene, has been identified as a potential biomarker for monitoring treatment response in advanced ovarian cancer. Its presence may also confer sensitivity to FGFR2-targeted therapies such as BGJ398 [29]. Similarly, BRCA1/2 reversion mutations can be identified through ctDNA analysis, providing valuable insight into resistance mechanisms and aiding in the selection of candidates for platinum-based chemotherapy or PARP inhibitor therapy [30].

From a prognostic standpoint, ctDNA-based detection of specific gene alterations has been associated with poorer PFS across histologic subtypes. For instance, TP53 mutations are commonly observed in high-grade serous carcinoma, APC in clear cell carcinoma, PIK3CA in endometrioid carcinoma, and KRAS in mucinous carcinoma [28]. Several studies have identified ctDNA as an independent prognostic marker for OS and recurrence risk in ovarian cancer. For example, Morikawa et al. detected PIK3CA-H1047R and KRAS mutations in tumor tissues from patients with clear cell ovarian cancer and successfully matched these alterations to ctDNA, which was longitudinally monitored throughout the study [31]. Mutant ctDNA levels were found to correlate with both disease recurrence and response to therapy [31]. However, these findings were not consistently reproduced. In a separate study, Ogasawara et al. reported that, while the detection of PIK3CA and KRAS mutations in ctDNA was associated with advanced-stage disease, it did not correlate with histologic subtype or residual tumor burden [32], underscoring the need for further research to clarify the prognostic implications of ctDNA across different ovarian cancer subtypes.

To further explore the prognostic gene signatures associated with clinical outcomes, Gunderson et al. utilized ctDNA to distinguish between patients with poor prognosis (progression-free interval < 6 months) and those with favorable prognosis (progression-free interval > 24 months) [33]. The study identified 29 genes amplified at higher levels in poor-prognosis patients, many of which are implicated in pathways related to cell invasion and metastasis. These findings suggest that ctDNA profiling may be a valuable tool in the development of targeted therapeutic strategies, enabling personalized treatment approaches based on the tumor’s molecular characteristics [33].

Beyond its role in tumor profiling, ctDNA has demonstrated promise in detecting disease recurrence and minimal residual disease with greater specificity and sensitivity than conventional markers, such as CA125 or CT imaging. Zhang et al. investigated the use of ctDNA as a surrogate marker for molecular residual disease following primary debulking surgery or adjuvant chemotherapy in ovarian cancer patients [34]. Their findings demonstrated that ctDNA outperforms CA125 in predicting disease relapse. While the study does not advocate for the complete replacement of CA125 in surveillance, it supports a complementary approach that incorporates ctDNA to enhance diagnostic accuracy and reduce the ambiguity often associated with the low sensitivity of CA125 alone [34]. Supporting this approach, additional studies in other cancer types have shown that ctDNA can predict recurrence up to seven months earlier than conventional imaging modalities, such as CT [35], highlighting its potential to facilitate earlier and more informed treatment interventions.

In addition to early detection, ctDNA also offers potential value in assessing disease burden at the initiation of treatment. In a study evaluating ctDNA dynamics in patients with metastatic ovarian cancer undergoing chemotherapy, Cartaxo Alves et al. found that elevated ctDNA levels following the first treatment cycle were associated with improved PFS compared to patients with stable or declining levels [26]. The authors attributed this phenomenon to the chemotherapy-induced apoptosis of tumor cells—particularly in cases of high disease burden—suggesting that a transient ctDNA surge may reflect heightened tumor sensitivity to treatment and, by extension, better clinical outcomes [26]. The utility of serial ctDNA monitoring in detecting minimal residual disease and predicting clinical outcomes in ovarian cancer has also been explored. Heo et al. assessed ctDNA levels at baseline—defined as initial diagnosis or surgery—and at three-month intervals thereafter. Their study demonstrated that the trajectory of ctDNA from baseline to six months was predictive of PFS [36]. Specifically, patients with persistent pathogenic mutations at follow-up exhibited significantly shorter PFS compared to those whose mutations became undetectable [36]. However, as ctDNA trends can be influenced by various biological and treatment-related factors, caution is warranted when interpreting these dynamics in clinical practice. Misinterpretation may lead to premature discontinuation of effective therapy, or the unnecessary continuation of aggressive treatment regimens.

In summary, ctDNA offers the dual advantage of early recurrence detection and comprehensive tumor profiling, enabling more personalized treatment strategies for ovarian cancer. Currently, commercial ctDNA testing for ovarian cancer is covered by Medicare. Despite its promising role in prognostication and surveillance, ctDNA remains a relatively novel modality, with its clinical utility currently limited by technical variability and interpretive challenges inherent to its recent adoption in oncology practice [25]. Longitudinal study designs may assist in identifying appropriate timepoints to discontinue serial ctDNA monitoring in patients demonstrating sustained clearance, thereby reducing unnecessary medical costs. Additionally, further research is warranted to investigate ctDNA dynamics in the context of combination therapies, particularly with the integration of novel agents, such as immune checkpoint inhibitors.

## 4. Expression of Therapeutic Biomarker in Ovarian Cancer

As previously mentioned, ovarian carcinomas are often diagnosed at advanced stages (stage III–IV), with surgical debulking and chemotherapy being the mainstays of treatment. Various histologic types of epithelial ovarian cancers, however, have different sensitivities to chemotherapy. For example, the overwhelming majority of HGSOC patients respond well to initial chemotherapy with carboplatin and paclitaxel, although the majority will ultimately experience recurrence.

With the recent advent of tumor genomic profiling, various prognostic and therapeutic biomarkers have been discovered that allow for a more personalized treatment approach. Here, we will discuss key biomarkers with clinical relevance in ovarian cancer, namely breast cancer susceptibility genes 1 and 2 (BRCA), human epidermal growth factor receptor 2 (HER2), trophoblast cell surface antigen 2 (TROP2), and folic acid receptor alpha (FOLR1). Additionally, we will discuss their implications for personalized therapy and targeted treatment strategies. An overview can be seen in Table 1.

### 4.1. Breast Cancer Susceptibility Genes (BRCA1/2)

Germinal mutations in the BRCA genes confer a lifetime risk of ovarian cancer as high as 70%. These cases represent about 10% of ovarian cancer diagnoses. BRCA1 and 2 are part of the tumor suppressor gene family, located on chromosomes 17 and 13, respectively, and play critical roles in homologous recombination DNA repair. While BRCA mutations confer risks of multiple other cancers in a syndromic fashion, these biomarkers are characterized by favorable responses to platinum-based chemotherapy and targeted maintenance strategies, like poly (ADP-ribose) polymerase (PARP) inhibitors. The first PARP inhibitor studied was olaparib, through the SOLO1 trial [37]. This trial tested the use of olaparib as a maintenance treatment after complete or partial response to standard platinum-based chemotherapy for BRCA-mutated, stage III/IV high-grade serous or endometrioid ovarian cancers. The Median PFS after 5 years of follow up was 56 months in the olaparib arm, versus 13.8 months in the placebo arm. Additionally, the median OS after 7 years of follow up was not reached in the olaparib arm versus 75.2 months in the placebo group [38]. After the success of SOLO1, additional clinical trials for niraparib (PRIMA) and rucaparib (ATHENA) were published, further demonstrating the activity of PARP inhibitors for the treatment of BRCA-mutated ovarian cancer patients [39]. For olaparib, the current FDA-approved indications include first-line maintenance treatment following response to platinum-based chemotherapy for newly diagnosed, advanced-stage, high-grade ovarian cancer patients harboring germline or somatic deleterious BRCA alterations, in combination with bevacizumab in the presence of germline or somatic deleterious BRCA alterations and/or homologous recombination-deficient (HRD) tumors [40].

### 4.2. Human Epidermal Growth Factor Receptor 2 (HER2)

HER2 is a receptor tyrosine kinase that is encoded by the ERBB2 gene. If ERBB2 is amplified, HER2 overexpression and receptor dimerization are constitutively activated in the cell, leading to persistent signaling of the mitogen-activated protein kinases and the PI3K pathway [41]. These pathways ultimately control the cell cycle, cell metabolism, and cellular differentiation [42]. HER2 expression has been studied in both high- and low-grade serous ovarian carcinomas, with reported expression levels noted to be between 5 and 60% [43,44,45]. HER2-overexpression represents a poor prognostic marker in multiple tumors, including ovarian cancer, where it is associated with chemoresistance, poor OS (HR = 1.57, 95%CI = 1.31 to 1.89), and decreased PFS (HR 1.26, 95%CI = 1.06 to 1.49) [44]. HER2 expression levels can be evaluated by immunohistochemistry (IHC). Given the differences in HER2 staining between breast cancer (circumferential) and gynecologic malignancies (basolateral positivity with apical sparing), different IHC scoring algorithms have been proposed [46]. The following studies used a method similar to gastric staining methods; however, some pathology labs have incorporated IHC with directed FISH testing of areas of expression, with those that tested HER2 2+- and FISH-positive considered for HER2-directed therapy along with those that are HER2 IHC 3+. HER2 expression can also be identified through the sequencing of tumor tissue, noted as ERBB2 amplification.

HER2-directed treatment in gynecologic malignancies, including recurrent ovarian cancer, was evaluated after its successful use in breast cancer patients overexpressing HER2 [47]. Ovarian cancer-targeted treatment was initially based on the use of unconjugated antibodies such as trastuzumab and pertuzumab [48,49]. However, limited efficacy was demonstrated with the use of “naked” antibodies targeting HER2 platinum-resistant or recurrent ovarian cancers [48,49]. Accordingly, in the last few years, driven by their higher clinical activity when compared to unconjugated antibodies and improved efficacy compared to traditional chemotherapy, the use of antibody–drug conjugates (ADCs) targeting HER2 has rapidly expanded against a variety of human tumors, including ovarian, uterine, and cervical cancers. Consistent with this view, trastuzumab emtansine (T-DM1), was developed using the monoclonal trastuzumab linked with a non-cleavable linker to the toxic payload emtansine, a microtubule inhibitor [50]. T-DM1 was studied in breast cancer and was found to have an objective response rate (ORR) of 33% and mean duration of response (DOR) of 9.7 months [51]; however, in ovarian cancer, the ORR was lower (i.e., one out of seven patients, or 14%) [51]. The KATHERINE trial eventually led to the FDA approval of T-DM1 in May 2019 for HER2-positive breast cancers, as it showed improved invasive disease-free survival compared to use of trastuzumab alone [52].

Another ADC, trastuzumab deruxtecan (T-DXd) was more recently developed using the trastuzumab monoclonal antibody connected to the highly potent toxic payload deruxtecan (a topoisomerase I inhibitor) with a cleavable linker [53]. This cleavable linker is degraded by cathepsins B and L, and, once internalized, it can diffuse through the cell membrane to neighboring cells, inducing the bystander effect and treating neighboring cells that may not express HER2 [54]. Preclinical studies of T-DXd have shown that HER2 overexpression allows for higher sensitivity to T-DXd than lower expression [55]. T-DXd was first studied in gynecologic malignancies with the STATICE trial [56]. This study showed an ORR of 54.5% in highly expressing tumors, and 72% ORR in the low-expressing group. Interestingly, disease control was 100% regardless of HER2 expression level. Ultimately, T-DXd was studied in the DESTINY-PanTumor02 trial, a Phase II study, in patients with HER2-expressing solid tumors, including ovarian cancer. In ovarian cancer, the trial showed an overall response rate of 45%, with 63.6% in patients with HER2 IHC 3+ expression. These clinical trial results granted T-DXd an agnostic FDA approval indication for all solid tumors with HER2 positivity (3+ expression) in April 2024 [57].

An additional HER2-targeting ADC (i.e., Synthon-985, trastuzumab duocarmazine), was developed by Synthon/Blondis (Nijmegen, The Netherlands). Synthon-985 was also based on the linking of trastuzumab to a highly cytotoxic prodrug, seco-duocarmycin-hydroxybenzamide-azaindole (seco-DUBA) [58]. Using duocarmazine as a toxic payload, this ADC functions by binding to the minor groove of DNA, causing selective alkylation of N3 adenosine residues [58]. With this mechanism, a 3–50-fold greater cytotoxic effect is seen in comparison to T-DM1 in HER2 low-expressing cells [59]. While we currently lack clinical trial data for trastuzumab duocarmazine in ovarian cancer, preclinical data in this setting are promising; as the ADC remains stable in circulation, it is endowed with a potent bystander effect, and it is not only more potent than T-DM1, but is also able to trigger cytotoxicity in non-replicating tumor cells [60].

Aside from being a prognostic biomarker and therapeutic target, HER2 also interplays with noncoding RNAs that, when dysregulated, can alter the expression levels of HER2 and, ultimately, lead to chemoresistance and progression in ovarian cancer. Yang et al. [61] demonstrated that the miRNA miR-429 can target and downregulate the expression of key components of HER2 signaling, therefore restoring cell proliferation and regulation. However, additional preclinical work in this area is needed to potentially target these non-coding RNAs for HER2 manipulation and better treatment of ovarian cancer.

### 4.3. Trophoblast Cell Surface Antigen 2 (TROP2)

TROP2 is encoded by the TACSTD2 gene on chromosome 1p32 and is a transmembrane glycoprotein [62]. It plays important roles during cell proliferation and self-renewal, and it has been implicated in tumor cell invasion and metastasis [63]. TROP2 is expressed in the majority of ovarian cancers, with 42% of cases demonstrating strong expression, with moderate expression in 26.5% and weak expression in 29.8% by IHC. Only 1.8% have no expression of TROP2 in tumor cells [64]. TROP2 overexpression is a poor prognostic indicator and correlates with poor PFS and OS in ovarian cancer [65]. This transmembrane protein is preferentially expressed on tumor cells and is, therefore, an appropriate target for ADCs such as sacituzumab govitecan and datopotamab deruxtecan.

Sacituzumab govitecan is an ADC made up of a TROP2-targeting humanized antibody (hRS7) linked to a toxic payload, SN-38, an active metabolite of irinotecan that is 100-fold more potent than irinotecan itself [62]. These two components are connected by a cleavable linker, and the ADC has a drug–antibody ratio (DAR) of 7.6:1 [66]. Preclinically, sacituzumab govitecan has demonstrated remarkable success in suppressing tumor growth in lines that strongly express TROP2 [67]. Additionally, preclinical models have proven that the cleavable linker is able to induce bystander killing, which is advantageous in heterogenous ovarian cancers, where not all cells are strong expressors of TROP2 [67]. In February 2023, the FDA approved sacituzumab govitecan for use in locally advanced/metastatic, unresectable HR-positive HER2-negative breast cancers, on the basis of the TROPICS-02 trial, which saw improvement in PFS (5.5 months vs. 4 months) and OS (14.4 months vs. 11.2 months) when compared to single-agent chemotherapy [68]. Clinical trials are currently ongoing, with the use of sacituzumab govitecan against multiple gynecologic malignancies including ovarian cancer (i.e., NCT06028932), in an open-label, non-randomized, Phase II clinical trial, enrolling patients with platinum-resistant ovarian cancer. Early reports from compassionate-use cases of this ADC showed promise in a heavily pretreated ovarian cancer patient [69].

Datopotamab deruxtecan (Dato-DXd) is another ADC using a TROP2-targeting antibody, also linked to a topoisomerase I inhibitor as a toxic payload. The use of a cleavable linker again in this ADC allows for stability in circulation, payload release, and the bystander effect, to allow for cytotoxicity in neighboring tumor cells that do not express TROP2 strongly [70]. Preclinically, Dato-DXd has proven to be an effective ADC to allow for the inhibition of tumor growth in strongly expressing cell lines. Additionally, antibody-dependent cellular cytotoxicity (ADCC) experiments with Dato-DXd have shown that this ADC can induce killing of ovarian cancer tumor cells, not only with the toxic payload, but also through the activation of natural killer (NK) cells binding to the Fc component of the IgG1 Ab [71]. While clinical trials with this ADC are still pending, it has the potential to be more effective than sacituzumab govitecan. Indeed, the toxic payload in Dato-DXd is 10 times more potent than SN-38 and has an extended half-life in comparison [72]. This allows for extended dosing in comparison to sacituzumab govitecan, which requires dosing on days 1 and 8 versus every 21 days. Dato-DXd has a smaller DAR in comparison to sacituzumab govitecan, allowing for decreased systemic toxicity and an optimized therapeutic window [70]. In January 2025, the FDA approved Dato-DXd for use in patients with unresectable or metastatic, HR-positive, HER2-negative breast cancer, based on the TROPION-Breast01 trial, which demonstrated a median PFS of 6.9 months versus 4.9 months and a median OS of 18.6 months vs. 18.3 months compared to standard chemotherapy [73]. The use of TROP2 as a biomarker for gynecologic malignancies is being rapidly studied, and further research into newly developed ADCs, such as sacituzumab tirumotecan (MK-2870), is underway.

### 4.4. Folic Acid Receptor Alpha (FOLR1)

Folate, also known as vitamin B9, is important for normal cell functions, such as the transport of one-carbon units for DNA and RNA synthesis, as well as in fetal neural tube development [74]. While vital for normal cellular functioning, tumor cells have hijacked this mechanism for use in tumorigenesis, as well [75]. The folate receptor exists in four different isoforms (alpha, beta, delta, and gamma); however, the alpha receptor has been studied the most. FR alpha is a membrane-bound glycosyl-phosphatidylinositol (GPI) that is encoded by the FOLR1 gene found on chromosome 11 [76]. The receptor has a high affinity for folate, as well as its reduced forms, and transports folate to the cytoplasm via potocytosis [77]. The downstream pathways of folate receptor alpha include the STAT3 pathway, which is important for regulating cell growth. In tumor cells, the function of this receptor also includes the downregulation of the adhesion molecule E-cadherin, allowing for tumor invasion and spread [78]. In ovarian cancer, specifically, folate receptor alpha has a role in the downregulation of the tumor suppressor caveolin-1 (cav-1), which is important for cell proliferation [79]. The folate receptor is found on tissues throughout the body; however, it is more highly expressed on various tumor cells, like head/neck cancers (>45%), breast cancers (>30%), lung cancers (>72%), GI cancers (>33%), endometrial cancers (>70%), and ovarian cancers (>80%) [77]. A recent publication from our lab revealed that, in low-grade serous ovarian cancers (LGSOCs), specifically, the expression of FOLR1 is as high as 100%, with 78% having moderate to high expression levels by IHC [80].

Mirvetuximab soravtansine was the first ADC created for targeting folate receptor alpha. It comprises the toxic payload of DM4, a maytansinoid that is a tubulin inhibitor [81]. Its use was studied in the SOYAYA study [82] and was approved by the FDA in November 2022 after the results of the MIRASOL trial showed a PFS of 5.62 months and ORR of 42.3% in the mirvetuximab soravtansine-treated group compared to chemotherapy with paclitaxel, liposomal doxorubicin, or topotecan [83]. This approval was for platinum-resistant ovarian cancer. The use of mirvetuximab soravtansine may be indicated, however, for other cancers, such as low-grade ovarian cancer. Consistent with this view, in a recent report, we have shown that folic acid receptor alpha is not only expressed in the majority of these rare tumors, but also demonstrated preclinical efficacy of this ADC in a LGSOC patient-derived xenograft (PDX) model, as well as in a heavily pretreated LGSOC patient [80]. Future clinical trials are warranted to fully evaluate the clinical activity of mirvetuximab soravtansine in LGSOC patients.

In addition to mirvetuximab soravtansine, folate–drug conjugates are also in development. The first of these is EC131, which has the maytansinoid DM1 linked to folic acid via an intramolecular disulfide bond [84]. Given the high affinity for the folic acid receptor, this agent has shown promising cytotoxic effects preclinically. Vintafolide (EC145), also an option, is a water-soluble folic acid derivative linked to desacetylvinblastine monohydrazide, a microtubule-destabilizing agent. Both of these are currently in phase I and II trials [84].

## 5. Discussion

Ovarian cancers remain the most lethal gynecologic cancers. Most ovarian cancer patients are diagnosed at advanced stages (stage III–IV), secondary to lack of effective screening strategies for early detection. CA125 has been demonstrated in multiple studies to be neither sufficiently sensitive nor specific enough to be used as a biomarker for screening of the general population [12]. At present, the National Comprehensive Cancer Network (NCCN) recommends monitoring with CA125 measurements, as well as CT scans, when there is a concern for recurrence [85]. CA125, however, can be elevated nonspecifically, secondary to inflammation or a variety of benign conditions. In the last few years, the use of ctDNA has emerged as a novel, highly specific strategy for the detection of residual, recurrent disease in cancer patients. As a form of liquid biopsy, ctDNA can be detected with high specificity after patient-specific alterations are identified with sequencing of tumor tissue (i.e., tumor-informed assay) [25]. Consistent with this view, several studies have found a correlation with ctDNA and the presence of disease, often times earlier than a CT scan will show recurrence [34,35]. Additionally, the quantity of ctDNA present directly after surgical debulking procedures is also indicative of residual disease and, ultimately, can confer a negative prognosis [34]. Liquid biopsy with ctDNA measurements represents a novel monitoring method for ovarian cancers; however, prospective studies are lacking, and larger trials are needed before ctDNA can be used as a standardized methodology in clinical practice.

While the mainstay of treatment remains chemotherapy (either neoadjuvant or adjuvant) and surgical debulking, the advent of the genetic testing of tumors has broadened the treatment options for ovarian cancer patients. One of the first biomarkers identified in ovarian cancer treatment was BRCA1/2. While an unfortunate diagnosis, as many patients with BRCA1/2 mutations are predisposed to develop multiple tumors, including breast cancer, its detection in ovarian cancer patients is paradoxically linked to a more favorable prognosis. Moreover, PARP inhibitors were developed and introduced in 2018 as maintenance medications, with improved survival outcomes in patients with BRCA1/2 mutations [37]. This approach, now considered the standard of care, has laid the path for the discovery and implementation of other biomarkers as therapeutic targets, including HER2, TROP2, and FOLR1, which are highlighted in this review.

HER2 is found to be highly expressed in many solid tumors, especially breast, gastric, and multiple gynecologic tumors. Unlike other malignancies, the expression of HER2 is not homogenous, and this has led to recent updates in pathology immunohistochemistry scoring for HER2 in gynecologic malignancies [86]. These characteristics in HER2 expression support the benefits of using an ADC for the targeted treatment of HER2-positive tumors over unconjugated antibody alone. The DESTINY-PanTumor trials have shown improved survival benefit and PFS patients harboring HER2-positive status by immunohistochemistry with the use of an ADC called trastuzumab deruxtecan (T-DXd) [57]. With its cleavable linker, trastuzumab deruxtecan has the advantage of inducing a bystander effect, allowing neighboring cells with lower HER2 expression to benefit from the ADC’s toxic payload. The benefits of this ADC were shown preclinically and clinically, ultimately leading to the agnostic approval of trastuzumab deruxtecan by the FDA for all HER2-expressing solid tumors.

TROP2 is one of the newest biomarkers to be studied in gynecologic malignancies. Multiple preclinical studies have shown a high expression level of TROP2 in ovarian cancers, and preclinical data on the use of targeted treatments, such as sacituzumab govitecan and datopotamab deruxtecan, have shown fantastic responses in cases overexpressing TROP2 [71,87]. Further ADCs targeting TROP2 are currently in development and testing and, if clinically successful, will broaden available options for patients. The first phase II trial with sacituzumab govitecan in ovarian cancer patients with recurrent platinum-resistant ovarian cancer has recently completed accrual in the USA [88].

Folate receptor alpha, encoded by FOLR1, is a biomarker targeted by multiple ADCs, either already approved (i.e., mirvetuximab soravtansine) or in advanced stages of development. Mirvetuximab soravtansine is currently approved for the treatment of FOLR1-positive (i.e., 75% 2+ or above), platinum-resistant ovarian cancer. Hence, while the ADC is now commonly used in clinical practice, high cut-off points significantly reduce the number of eligible patients for ovarian cancer treatment to about 30–35% [82]. Accordingly, lowering the cutoff for FOLR1 expression eligibility using novel, more potent ADCs targeting FOLR1 might significantly increase the number of patients who potentially benefit from this therapeutic target. Consistent with this view, multiple clinical trials with second-generation ADCs targeting FOLR1 in ovarian cancer patients are currently ongoing (Sutro Biopharma, Inc., San Francisco, CA, USA) [89].

A limitation to the adaptation of these biomarkers for therapeutic use is the reliance on the pathologic evaluation of most of these biomarkers with IHC testing of tumor samples in a reliable and efficient manner. While tests evaluating HER2 scoring have widespread use, smaller institutions may have limited access to testing for TROP2 and FOLR1. Unfortunately, many ADC studies in ovarian cancer and other gynecologic malignancies are limited to preclinical and clinical trial results, given how recently these agents have been produced and FDA-approved. Moreover, no comparative studies comparing one ADC to another in ovarian cancer have been published, with most of the ADC clinical protocols focusing on single-arm trials, or trials comparing ADCs to chemotherapy, rather than direct comparisons between different ADCs.

## 6. Conclusions

Ovarian cancer remains the most lethal gynecologic malignancy. The use of CA125 as a serum monitor continues to be the gold standard; however, ctDNA alone, or in combination with protein biomarkers, has recently emerged as a more specific and sensitive biomarker combination for the monitoring and detection of early recurrence in ovarian cancer patients. Routine genomic testing of tumor tissue has expanded the treatment options for ovarian cancer patients, with the discovery of biomarkers including, but not limited to, BRCA1/2, HER2, TROP2, and FOLR1, all providing successful second-line treatments for patients developing this aggressive form of gynecologic tumors.

## Figures and Tables

**Table 1:** Biomarkers in ovarian cancer. Depicted here are the various biomarkers discussed in this review, with their associated downstream effects in cancer biology and associated targeted therapeutics, either currently in development or in clinical use.

Biomarker	Downstream Effect	Targeted Therapeutic
BRCA1 or BRCA2 mutation	Deficient homologous recombination and DNA repair	PARP inhibitor (olaparib, niraparib, rucaparib)
HER2	Chemoresistance, decreased overall survival, and progression-free survival	Monoclonal antibodies (trastuzumab, pertuzumab); antibody–drug conjugates (T-DM1, T-DXd, trastuzumab duocarmazine)
TROP2	Increased invasive potential, decreased overall survival, and progression-free survival	Antibody–drug conjugates (sacituzumab govitecan, datopotamab deruxtecan, sacituzumab tirumotecan)
FOLR1	Decreased E-cadherin and Cav-1; increased invasive potential; increased cellular proliferation	Antibody–drug conjugates (mirvetuximab soravtansine), folate–drug conjugates (EC131, EC145)

## Data Availability

All data are referenced in-line for availability in the References section of this manuscript.

## Abbreviations

The following abbreviations are used in this manuscript:

HGSOC	High-Grade Serous Ovarian Cancer
HRD	Homologous Recombination Deficiency
TMB	Tumor Mutational Burden
TROP2	Trophoblast Cell-Surface Antigen 2
CA125	Cancer Antigen 125
GCIG	Gynecologic Cancer InterGroup
PFS	Progression-Free Survival
OS	Overall Survival
CT	Computed Tomography
ctDNA	Circulating Tumor DNA
VAF	Variant Allele Frequency
FGFR2	Fibroblast Growth Factor Receptor 2
BRCA	Breast Cancer Susceptibility Gene
HER2	Human Epidermal Growth Factor Receptor 2
FOLR1	Folate Receptor Alpha
PARP	Poly (ADP-Ribose) Polymerase
ADC	Antibody–Drug Conjugate
ORR	Objective Response Rate
DOR	Duration of Response
DAR	Drug–Antibody Ratio
GP1	Glycosyl-Phosphatidylinositol
LGOC	Low-Grade Serous Ovarian Cancer
NCCN	National Comprehensive Cancer Network
